# Utilizing Social Media to Identify Potential Living Donors: Learning from US Living Donor Programs

**DOI:** 10.1007/s40472-022-00382-1

**Published:** 2022-11-22

**Authors:** Angie G. Nishio-Lucar, Heather F. Hunt, Sarah E. Booker, Laura A. Cartwright, Lindsay Larkin, Stevan A. Gonzalez, Jessica A. Spiers, Titte Srinivas, Mahwish U. Ahmad, Macey L. Levan, Pooja Singh, Heather Wertin, Cathy McAdams, Krista L. Lentine, Randolph Schaffer

**Affiliations:** 1 Department of Medicine, University of Virginia, Charlottesville, VA USA; 2 LIVE ON Organ Donation, Inc., Longmeadow, MA USA; 3 United Network for Organ Sharing (UNOS), Richmond, VA USA; 4 Department of Medicine, Baylor Simmons Transplant Institute, Baylor Scott & White All Saints Medical Center, Fort Worth, TX USA; 5 Virginia Mason Transplant Center, Seattle, WA USA; 6 CareDx, South San Francisco, CA USA; 7 Center for Bioethics, Cleveland Clinic, Cleveland, OH USA; 8 New York University, New York, NY USA; 9 Department of Medicine, Thomas Jefferson University, Philadelphia, PA USA; 10 Barnes Jewish Hospital, St. Louis, MO USA; 11 Nemours/Alfred I. duPont Hospital for Children, Wilmington, DE USA; 12 Center for Abdominal Transplantation, SSM-Saint Louis University Hospital, St. Louis, MO USA; 13 Department of Surgery, Division of Organ Transplantation, Scripps Clinic, La Jolla, CA USA

**Keywords:** Living donation, Social media, Survey, Practices, Transplantation

## Abstract

**Purpose of Review:**

Living donor transplantation provides the best possible recipient outcomes in solid organ transplantation. Yet, identifying potential living donors can be a laborious and resource intensive task that heavily relies on the recipient’s means and social network. Social media has evolved to become a key tool in helping to bring recipients and potential living donors together given its ease of utilization, widespread access, and improved recipient’s comfort with public solicitation. However, in the USA, formal guidelines to direct the use of social media in this context are lacking.

**Recent Findings:**

To better inform the landscape and opportunities utilizing social media in living donation, the OPTN Living Donor Committee surveyed US transplant programs to explore programs’ experiences and challenges when helping patients use social media to identify potential living donors (September 2019). A large majority of survey participants (*N* = 125/174, 72%) indicated that their program provided education to use social media to identify potential living donors and most programs tracking referral source confirmed an increase utilization over time. The use of social media was compounded with program and recipient’s challenges including concerns about privacy, inadequate technology access, and knowledge gaps. In this review, we discuss the results of this national survey and recent literature, and provide suggestions to inform program practices and guidance provided to patients wishing to use social media to identify potential living donors.

**Summary:**

Transplant programs should become competent in the use of social media for potential living donor identification to empower patients interested in using this tool. Social media education should be provided to all patients regardless of voiced interest and, when appropriate, revisited at multiple time points. Programs should consider developing a “team of experts” that can provide focused education and support to patients embarking in social media living donor campaigns. Care should be taken to avoid exacerbating disparities in access to living donor transplantation. Effective and timely guidance to patients in the use of social media could enhance the identification of potential living donors.

**Supplementary Information:**

The online version contains supplementary material available at 10.1007/s40472-022-00382-1.

## Introduction

Patients in need of a life-saving organ transplant face an uncertain future. In the USA, organ wait times are usually long and variable depending on geography, organ type, and various patient factors [1–3]. Patients waiting for a deceased donor kidney transplant face particularly lengthy wait times [4, 5]. Living donor transplantation can offer patients timely and efficient access to transplant while usually providing better outcomes than deceased donor transplantation [6–9]. However, complex and multifaceted barriers exist that limit a patient’s ability to identify a potential living donor [10–13]. Some of these barriers arise from knowledge gaps about the risks and benefits of living donor transplantation, misinformation about the donation process, and, importantly, patient’s discomfort with public solicitation for potential living donors [14, 15].

Data suggest that patients with robust social networks are more likely to succeed at identifying potential living donors [13, 16]. Traditional methods to communicate the need for a living organ donor beyond the immediate social network (i.e., personal contact, announcements at work/church/social gatherings, billboards, flyers, signs, or newspaper advertisements) while helpful, usually provide limited reach and may be less accessible to disadvantaged communities [13, 17]. Over the last two decades, social media platforms have become increasingly popular and, for many, social media is the main source of information acquisition and dissemination. The inexpensive, accessible, and “detached” nature of social media make it an attractive way to share individuals’ needs and aspirations to a broad audience. Furthermore, social media potential living donor solicitation may be less emotionally taxing than other forms of public solicitation [18]. Common social media channels used for living donor identification campaigns include social networking sites like Facebook, microblogging sites like Twitter, and media sharing sites like Instagram or Reddit. From all of these, the most studied and most often recommended as a starting point is Facebook given its ease of use, free membership, and multimodal capacity of communication (i.e., video, chat, and blogging). Facebook also offers the capacity to create “Facebook Pages” (originally intended for brands within the platform) allowing people to connect without requiring a friend status (or being a contact) and “Facebook Groups,” where people can connect for a common cause or interest such as a living donor campaign [19].

There are several concerns when using social media for living organ donor campaigns which often stem from the lack of supervision, absence of guidelines, and uneven agency among potential users [20–22]. Data to assist transplant programs on effective patient-counseling practices or informing how to best resolve the challenges emerging from these campaigns (i.e., a sudden influx of potential living donor inquiries and candidates, staffing issues, and uneducated potential living donors) are scarce. Thus, improving our understanding of social media use to identify potential living donors could enhance its utilization and positively impact living donor transplantation. Between 2018 and 2020, the Organ Procurement and Transplantation Network (OPTN) Living Donor Committee embarked on a project to learn from US living donor programs experiences and challenges resulting from recipients and donors use of social media. The Committee surveyed living donor programs to develop a guidance document for transplant programs wishing to help recipients using social media to identify potential living donors and offered possible solutions to programmatic challenges [23]. In this manuscript, we discuss the key findings of this survey along with the available literature supporting the use of social media to identify potential living donors.

## The OPTN Living Donor Committee Survey

In September 2019, the OPTN Living Donor Committee invited US living donor programs to participate in a survey exploring their (1) social media education and resources; (2) program perceived or reported patient challenges; (3) programmatic concerns and alleged challenges; (4) practice changes in response to experienced challenges; and (5) program preparedness to handle a sudden influx of potential living donors from a social media campaign. The survey questions were a mix of multiple-choice questions, select all that apply (i.e., answers were not necessarily mutually exclusive), categorical questions, Likert scales, and some allowed participants to provide qualitative answers. Disclosing center name and OPTN region were optional to maximize survey response. The survey was electronically distributed to the OPTN Living Donor, Transplant Administrator, and Transplant Coordinator Committee members, posted publicly on the OPTN and UNOS websites, directly electronically mailed to program directors, transplant administrators, and quality directors of living donor programs, and a link to the survey was included in the September 2019 issue of the UNOS Transplant Pro eNewsletter. An IRB exemption was obtained from the US Department of Health and Human Services Health Resources and Services Administration (HRSA).

There were a total of 174 survey responses from at least 91 unique transplant programs, representing all OPTN regions (Table 1). Transplant center name was not available for 36.2% (*N* = 63) survey responses and 23.6% (*N* = 41) did not provide an OPTN region. Based of those disclosing their transplant program, the estimated response rate was at least 38.7% (91/235 active living kidney and/or liver donor programs at the time of the survey). Participants were predominantly from kidney transplant programs (70.1%, *N* = 122) and frequently used digital questionnaires to collect health information during the initial screening (58.6%, *N* = 102). Those using digital questionnaires felt these enhanced the process efficiency (85.3%, *N* = 87). Most kept record of the donor referral source (i.e., phone call, social media; 87.9%, *N* = 153) with 62% (*N* = 97) receiving referrals from social network sites (e.g., Facebook and Instagram) and 11.6% (*N* = 18) from donor membership sites. The referral volume through social media was variable with most respondents indicating 50 or fewer of such referrals in the past year and only a handful (*N* = 6) received more than 100 social media referrals in the past year. Yet, the number of social media referrals appeared to be on the rise with 61% reporting greater number of these referrals over the past year.

**Table 1:** Respondents’ characteristics^†‡^

Survey responses by OPTN regions	%	*N* = 174
Region 1	2.9	5
Region 2	11.5	20
Region 3	9.2	16
Region 4	6.9	12
Region 5	9.2	16
Region 6	4.0	7
Region 7	6.9	12
Region 8	4.0	7
Region 9	5.7	10
Region 10	7.5	13
Region 11	8.6	15
Not reported	23.6	41
Survey responses by type of living donor program	%	*N* = 174
Kidney	70.1	122
Kidney and Liver	23.0	40
Liver	5.7	10
Not reported	0.6	1
Other	0.6	1
Number of responses per center	%	174
1 response(s)	41.4	72
2 response(s)	10	18
3 response(s)	0.6	1
Center not reported	36.2	63
Uses electronic or online donor intake questionnaire	%	*N* = 174
Yes	58.6	102
No	41.4	72
Type of electronic tool^†^	%	102
MedSleuth/Breeze	28.4	29
Internal Form/System	22.6	23
DASH/NKR	13.7	14
One Medical Passport	11.8	12
Other	19.6	20
Not reported	3.9	4
Tracks potential living donor referral route	%	*N* = 174
Yes	77.9	153
No	12.1	21
Potential living donor referral route^‡^	%	*N* = 153
Patient referral/directed donor	96.8	150
Social network sites (i.e., Facebook, Instagram)	62.6	97
Print media created by patient/families (i.e., flyers, billboards, posters, brochures)	53.5	83
Broadcast media created by patient/families (i.e., radio, television)	43.9	68
Media created by the living donor program	37.4	58
Online membership sites	11.6	18
Other	5.2	8
Change in potential living donor social media referrals^†^ in the last year	%	*N* = 100
Increase	61	61
Decrease	3	3
Unchanged	9	9
Don’t know/not reported	27	27

^†^Selected questions include only those answering “yes” as the denominator

^‡^For “select all that apply” type questions, the number of responses may exceed the number of survey participants

## Social Media Education and Its Impact on Transplant Programs

**Fig. 1:**
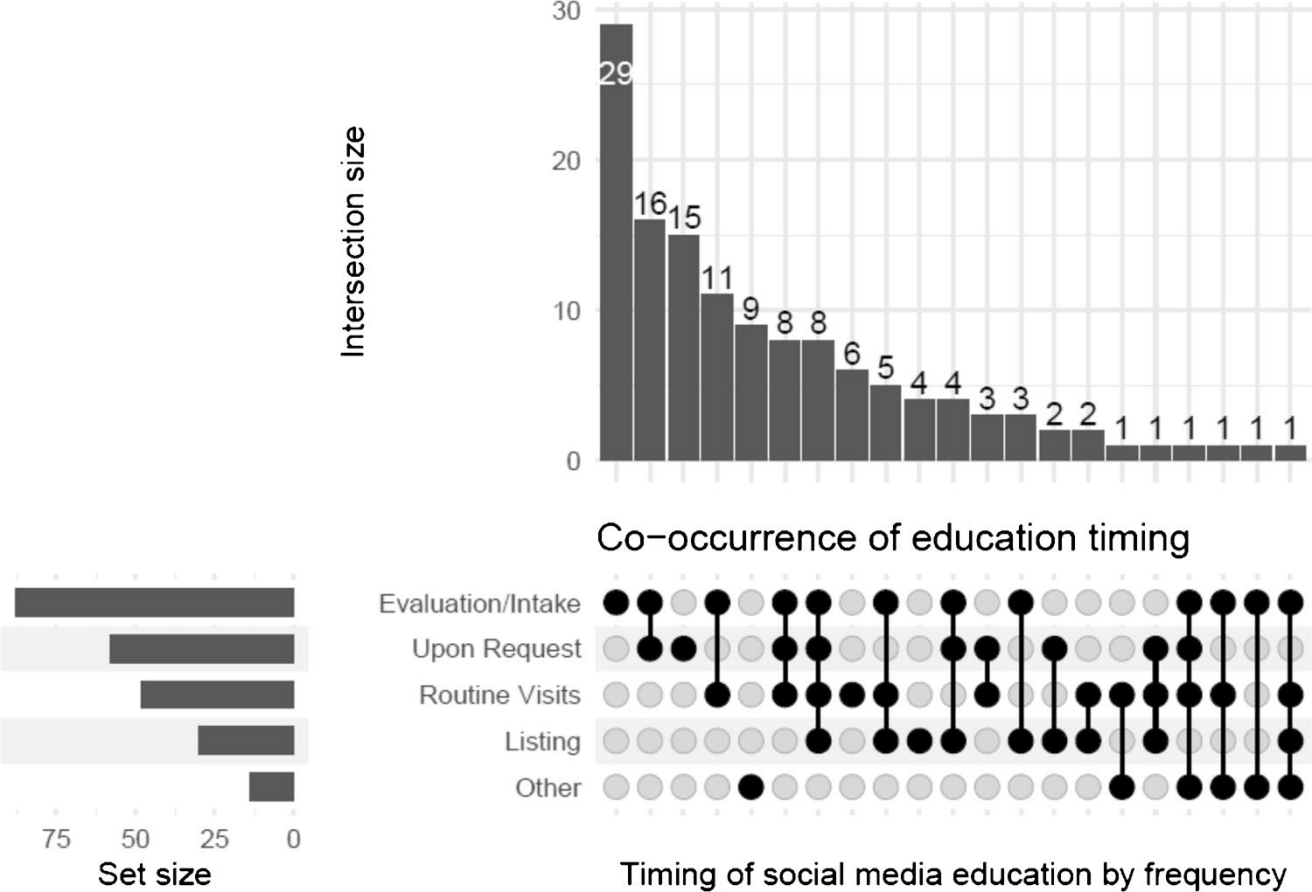
Co-occurrence of timing of social media education. The most common combination was at evaluation/intake and upon request (intersection size *N* = 16)

Most transplant programs were reported to provide social media education to identify potential living donors (78.2%, *N* = 136) but practices, timeline, and format were variable. Findings are summarized in Table 2. Discussions about the use social media in this context happened usually during the evaluation/intake (64.7%, *N* = 88) and was reinforced at multiple timepoints and upon request (Fig. 1). A few participants noted their program only offered such education when patients inquired about it. Clinical teams (74.3%, *N* = 101), social workers (33.1%, *N* = 45), and the independent living donor advocate (ILDA) (28.7%, *N* = 39) were the most frequent team members educating patients in this topic and typically on a one-on-one setting (64.7%, *N* = 88). Only 14.7% (*N* = 20) survey participants stated their center included a patient’s family and friends during this education. Survey participants also noted that discussing expectations and possible concerns from patient’s use of social media was important. Concerns about efficacy and efficiency of a given social media campaign, privacy and legal issues, and possible associated cost/expenses were cited (Table 2).

**Table 2:** Characteristics of social media education delivery^†^

Provides education about social media	%	*N* = 174
Yes	78.2	136
No	21.3	37
Not reported	0.6	1
Setting	%	136
1:1 counseling/education	64.7	88
Living donor champion program	43.4	59
Program specific written material	41.9	57
Live group class/training session	41.2	56
Third party written material	26.5	36
Third party websites	21.3	29
Support network education	14.7	20
Other	5.9	8
Timing	%	*N* = 136
During evaluation/intake	64.7	88
Once decided to list a candidate	22.1	30
During routine visits	35.3	48
Upon request	42.6	58
Multiple timepoints	50.0	68
Other	10.3	14
Type of educating provider	%	*N* = 136
Clinical team	74.3	101
Social worker	33.1	45
Independent living donor advocate	28.7	39
Social media/communications staff	2.9	4
Other	22.1	30
Type of specific expectations discussed^†^	%	*N* = 104
Likelihood of finding a donor	65.4	68
Privacy concerns	65.4	68
Legal concerns	13.5	14
Cost	4.8	5
Other	21.2	22

^†^Selected questions include only those answering “yes” as the denominator

**Fig. 2:**
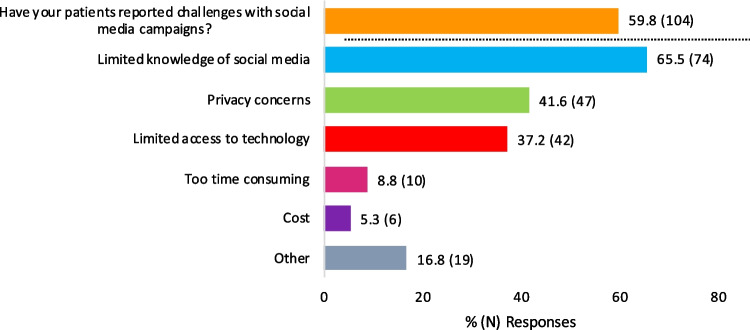
Patient-specific challenges from social media use. These challenges were reported by survey respondents; patients were not directly interviewed

**Fig. 3:**
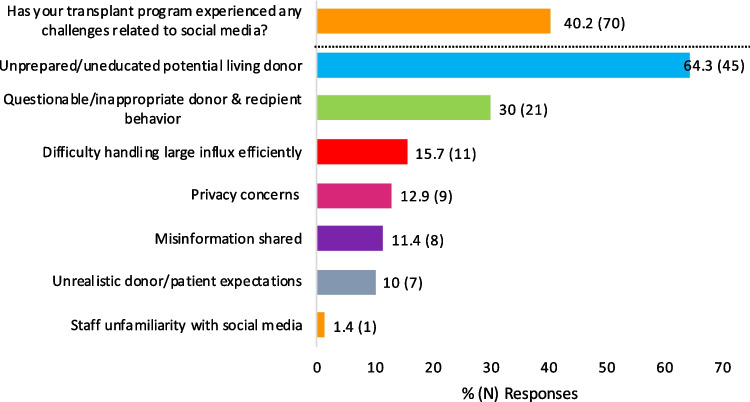
Programmatic challenges from patient’s living donor social media campaigns. Depicted themes were identified from qualitative responses. Collectively, they create awareness of potential program constraints

As expected, survey participants noted many patients experienced challenges when developing living donor social media campaigns which included limited know-how, privacy concerns, and limited access to technology (Fig. 2). Descriptive answers further revealed concerns about maintaining virtual engagement of potential living donors, fear of being a burden, and absence of guidance. At the program level, participant-reported challenges could be grouped in four main themes: referral volume, potential donor motivation, potential donor viability, and patient privacy. Many (40.2%, *N* = 70) felt that patient-implemented living donor social media campaigns lead to a higher number of unprepared/uneducated potential living donors and/or donor-recipient questionable/inappropriate behaviors (Fig. 3). Administrative strain was often cited, commonly in the form of high-volume donor inquiry (i.e., > 100 potential living donors stepping forward for a single patient in a short timeframe), insufficient staff and/or resources, and limited donor engagement with the evaluation process. To offset some of these issues, programs tailored the counseling provided to patients seeking to use social media and enhanced the screening questions and the evaluation of social media potential living donors. Frequently, cited practices include the following: (1) requiring a psychologist or psychiatrist evaluation prior to proceeding with other elements of the living donor assessment, (2) performing a social worker and/or ILDA assessment prior to appraising their medical candidacy, (3) applying “two-tier” screening questions with the second tier specifically inquiring about donation motives and examining for the presence of “secondary gain,” and (4) setting a minimum age to allow a social media donor to progress through the living donor evaluation. For many of the participant’s programs, these enhanced evaluation steps resembled those of a non-directed donor. A minority of participants stated that their evaluation process was no different for social media donors or non-directed donors.

## Social Media Learning Curve

**Fig. 4:**
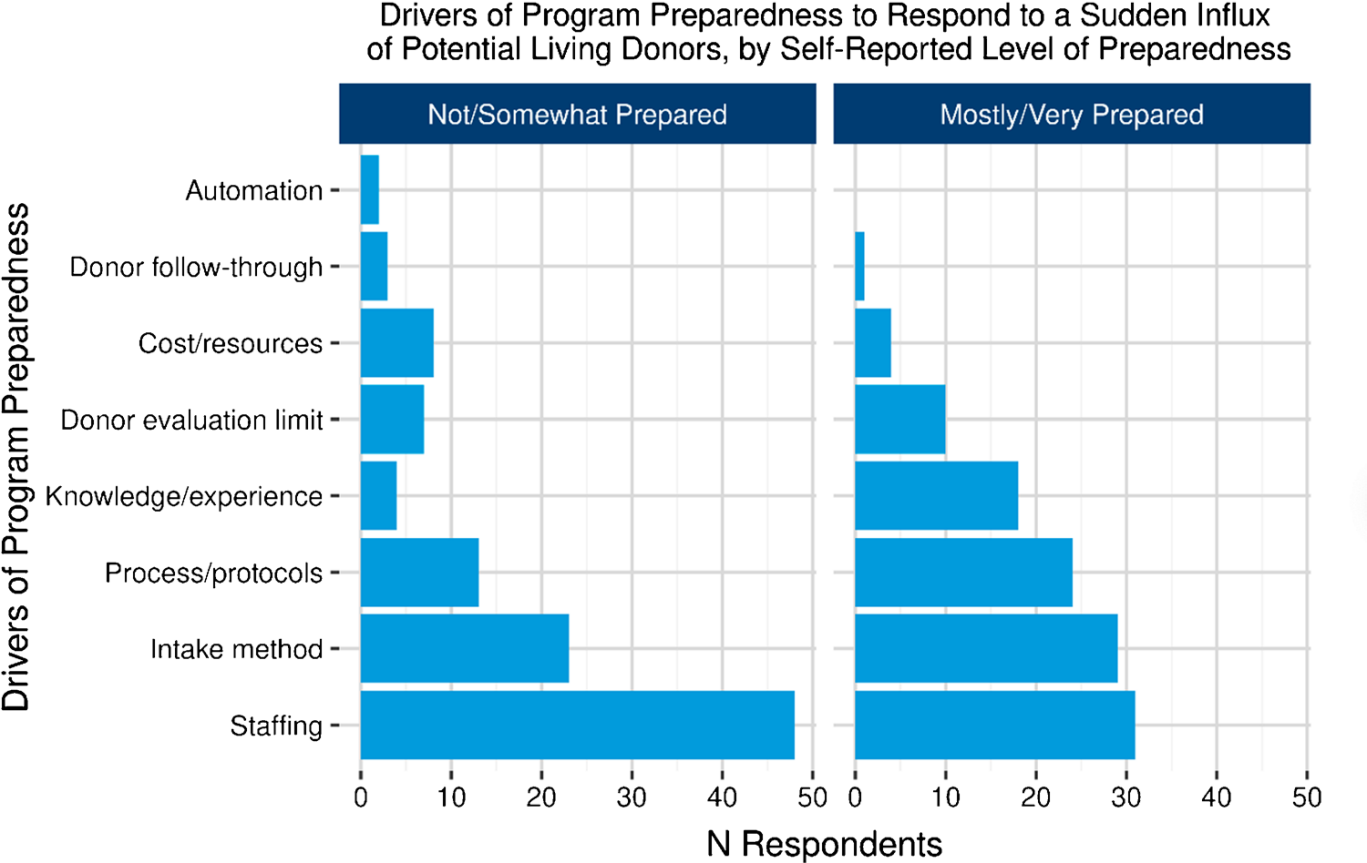
Living donor program level of preparedness. Describes living donor program’s attributes that facilitate the program’s preparedness

**Fig. 5:**
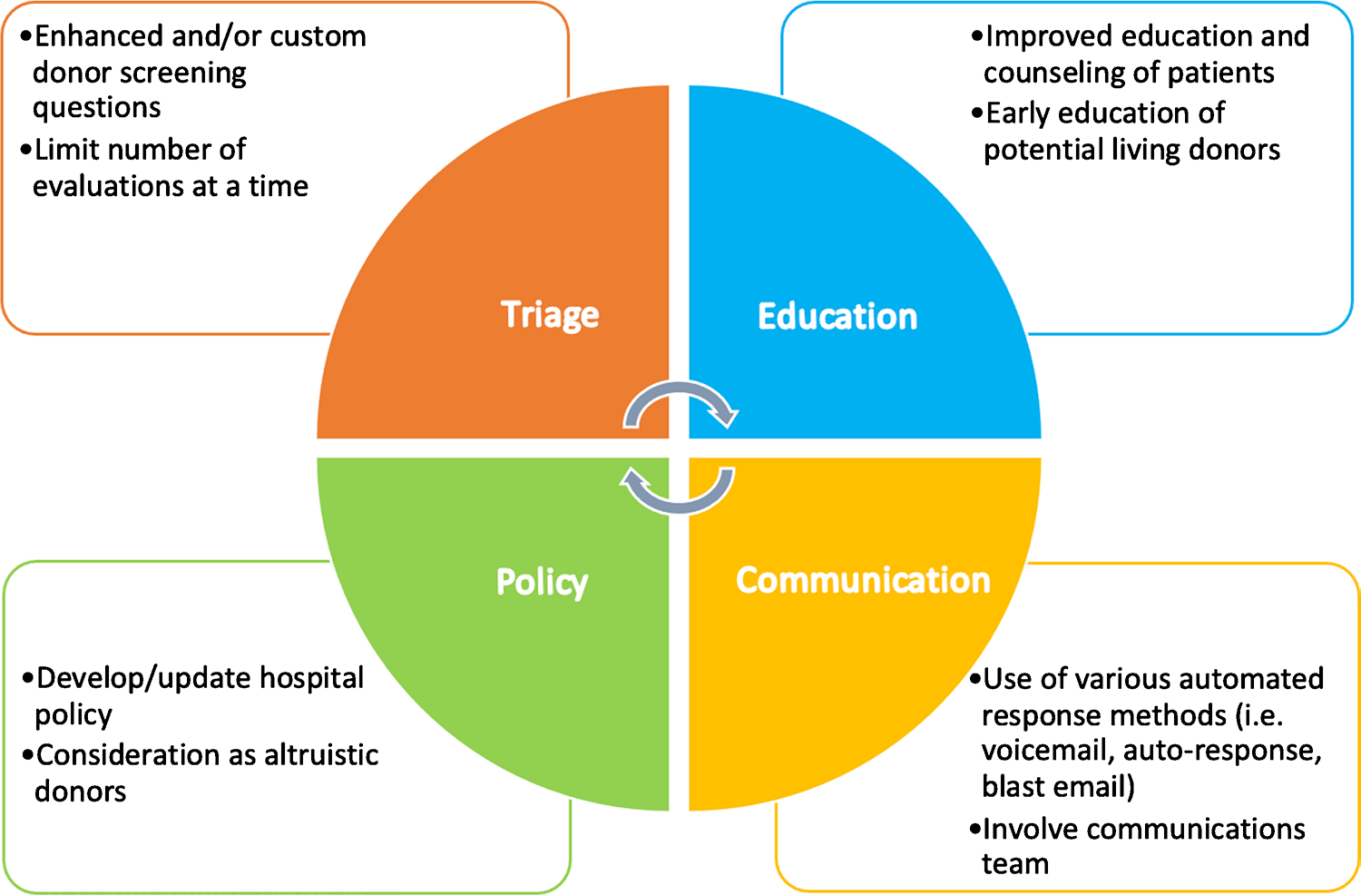
Living donor program practices to manage potential living donors referred through social media. There are 4 spheres of interventions that can enhance the evaluation of this type of donors

The survey identified some encouraging practices that could aid transplant programs welcome patient-driven social media living donor campaigns. Intuitively, greater familiarity with social media and routine acceptance of social media living donors among transplant programs correlated with greater awareness of patient-reported challenges (77.3% vs. 54.4%; *p* = 0.0128) and programmatic challenges (71.3% vs. 43.2%; *p* = 0.0031) which presumably leads to a more resourceful program when dealing with such challenges. There were eight transplant programs attributes reported to enhance a program’s level of preparedness to handle donors referred through social media that highlighted the importance of having experienced and/or adequate number of staff and streamlining the donor intake processes as key features driving preparedness (Fig. 4). However, having a social media living donor referral protocol, though believed to be useful, was unusual (79.9% *N* = 135 did not have protocols in place) and did not correlate with a program’s perceived preparedness to handle these donors (*p* = 0.3941). A qualitative analysis of 60 responses uncovered four spheres within a potential living donor evaluation process (triage, education, communication, and policy) where specific interventions could improve the process navigation (Fig. 5). Notably, an enhanced potential living donor assessment with custom questions and more detailed counseling for patients and families were deemed to be meaningful. Survey participants also noted that strategies such as limiting the number of simultaneous social media potential living donor evaluations per patient, along with strategic donor prioritization based on compatibility, donor engagement, donor-recipient relationship, and geographic location, were not only important but could ultimately necessitate patient involvement. Lastly, most survey participants (78.7%) reported their program commonly counseled incompatible social media living donors to consider non-directed donation instead.

## What Can We Learn About Use for Social Media for Living Donor Identification

This national survey of US transplant programs provides unique insight into the experiences of living donor programs stemming from patient-implemented living donor social media campaigns. Despite some methodological limitation discussed subsequently, there are several important findings worth highlighting. Living donor programs have demonstrated greater utilization and comfort with patient-implemented social media campaigns to identify potential living donors even in the absence of formal guidelines. Approximately three out of five participants in this survey reported that their program accepted potential living donor referrals through social media. Close to 80% routinely provided some form of education to patients in this topic and those tracking referral sources witnessed a rise in the number of social media referrals over the past year. This upward trend correlates with the increasing utilization of social media by transplant professionals as highlighted in a 2017 survey of members of the American Society of Transplant Surgeons reporting that 83% used social media for personal, professional, or patient/donor education purposes [24].

A recent study by Dubray et al. found that social media use to solicit potential living donors was not only prevalent but represented the majority of self-referrals to a single transplant center [25]. Social media potential living donors were more likely to be younger and more often exhibit directed altruistic intend compared to other routes of potential living donor solicitation. In fact, directed altruistic potential living kidney donors were almost exclusively generated through social media [25]. Another study by Novogrodsky et al. explored the effect of multiple types of media in non-directed living donors decision to donate, and reported that more than half (57%) of these donors identified the media as the cause for their initial interest in donation [26]. The majority stated that patients’ stories and personal narratives influenced their decision to donate [26]. Thus, social media could enable motivated altruistic individuals to become potential living donors and enhance living donor transplantation by connecting donor-recipient pairs who may not have been able to engage otherwise.

Despite the ubiquity of social media use in living donation, this survey noted considerable differences in how programs educated and counseled patients seeking to implement living donor social media campaigns. The timepoint and timeline of the education, the resources used (verbal, printed or digital media from within the program or outsourced), and setting of the education were highly variable. Only a small fraction (14.7%) of respondents reported including a patient’s family and support network when educating patients about social media in living donation. This finding contrasts with the recommendations from the 2014 AST Consensus Conference on Best Practices in Live Kidney Donation and other data suggesting that effective patient education should include a patient’s “family and friends” [13, 27, 28]. As such, programs who are already (or considering) educating patients about social media use should attempt to involve a patient’s network.

Notably, the OPTN survey found that potential living donors emerging from social media campaigns were often unprepared or insufficiently educated or had questionable/unrealistic expectations from the patient or the transplant program. While the level of donor unpreparedness was not precise, practices that could allow programs to overcome such challenges were offered. These include having experienced staff, automation of the donor intake process, and sensible donor prioritization which included limiting the number of donor evaluations occurring simultaneously. While a number of “manageable simultaneous potential living donor evaluations” was not quoted by survey participants, a recent publication by Habbous et al. discussing the cost-effectiveness of concurrent multiple living kidney donor evaluations for the same patient rather than sequentially, noted that that number may be up to four simultaneous potential living donor evaluations [29]. In Habbous’ study, limiting the number of evaluations to 4 concurrent ones leads to cost savings per intended recipient (despite the greater aggregate costs of multiple living donor evaluations) and increased living donor kidney transplant by 1% [29]. Habbous findings may serve as a reference for transplant programs wanting to prioritize potential living donor evaluations for a single recipient.

Since 2003, several social media sites have been launched offering various types of technology and capacity to share information [19, 30]. Social media is now embedded in most aspects of our everyday life, with platforms such as Facebook being used by most adult Americans on a daily basis [31, 32]. Pre- and post-kidney transplant patients are not different. Kazley et al. found that the majority of patients attending a single-center Renal Access Clinic (133 kidney transplant recipients and 66 transplant candidates) routinely used social media sites, with about one-third reporting more than 100 friends in their social media network and willing to post information about living kidney donation and/or their health status [33]. In the context of the COVID-19 pandemic, transplant programs found themselves reducing face-to-face interactions, limiting companions during visits, and rapidly transitioning to telemedicine encounters. These necessary adaptive changes to a “new normal” reshaped the role of social media and the transplant community relationship with digital technology [34, 35]. We have learned that technology can enhance our reach and empower transplant providers and patients to maintain (and likely gain) access to essential transplant. Although not uniform in content, several examples of websites offering guidance on how to use social media to identify potential living donors currently exist. A non-comprehensive list can be found in the supplemental material section (supplement 1).

As conveyed by survey participants, several ethical concerns arise from the use of social media in the context of living donation. Commonly cited concerns in the literature include the risk of compromising personal privacy and safety, maintaining veracity and truthfulness, interference with legal requirements necessary in living donation, coercive donor and recipient relationships and, inadvertently, worsening existing disparities in access to living donor transplantation [36, 37]. As an example, social media may favor patients possessing certain characteristics perceived to have a greater appeal (i.e., “beauty pageant effect” [38]), and disadvantage patients with limited resources and limited digital literacy [39]. Such concerns highlight the need for transplant professional societies and other stakeholder organizations to create guidelines that enhance the use of social media in living donation while safeguarding patients and potential living donors and fostering equitable access to this resource. A precedent for such guidelines was published by the Canadian Society of Transplantation in 2016 supporting the consideration of potential living donors from public solicitation, including social media, as legally and ethically acceptable [40]. The notion of public solicitation living donors being ethically reasonable is also supported by the American Medical Association’s code of medical ethics [41]. Future research should monitor the long-term psychosocial outcomes of living donors with diverse relationships with their recipients, including those identified through social media.

It is important to mention that data obtained through this national survey had some limitations. Namely, survey participants were not expected to identify their transplant program or OPTN region in an attempt to maintain anonymity. This issue results in the possible inclusion of multiple responses for the same transplant program or obtains information detailing specific program’s practices and regional trends. As responses to all questions were not mandatory, many questions were left unanswered and therefore data is not inclusive. Most importantly, two major events occurred after this survey took place. First, the CMS Conditions of Participation Interpretive Guidelines now mandate that the ILDA must interview potential living donors before a living donor evaluation can be initated [42]. This requirement may impact the living donor evaluation process efficiency and potentially diminished the enthusiasm about patient-implemented social media campaigns due to an increased process burden. Second and perhaps more importantly, this survey was administered before the COVID-19 pandemic and program practices could have changed since.

## Conclusion

Recognizing the influential nature of social media and its capacity to augment living donation rates, many thought-provoking questions remain unanswered. Do we need legislative and regulatory changes as social media gains traction in living donation? Should the transplant community collaborate with industry and communication experts to make this tool effective and accessible? How do we safeguard vulnerable groups from unintentionally disadvantaging them? And, should the use of social media be prioritized in social groups with lower rates of living donation? If so, is this an ethical practice? And, how do we measure its effectiveness and unexpected consequences? We must pay special attention to these challenges and proactively engage in providing guidance for safe and constructive use of this tool. In the end, social media and digital technology are here to stay so we will do well to embrace them in the service of optimizing transplant opportunities for our patients.

## Supplementary Information

Below is the link to the electronic supplementary material.

Supplementary file1 (DOCX 14 KB)
