# Supplementary material for: Utilizing Social Media to Identify Potential Living Donors: Learning from US Living Donor Programs
Source: Curr Transplant Rep. 2022 Nov 22;9(4):318–27. doi: 10.1007/s40472-022-00382-1 (PMC9684893; doi:10.1007/s40472-022-00382-1)
Supplement: Supplementary file 1 — Supplementary file1 (DOCX 14 KB) [file 40472_2022_382_MOESM1_ESM.docx]

| Organization | Website |
| --- | --- |
| National Kidney Foundation (NKF) | “How to Share Your Story and Find a Living Kidney Donor”  <https://www.kidney.org/newsletter/how-to-share-your-story-and-find-living-kidney-donor> |
| United Network of Organ Sharing (UNOS) | “How to find a living donor: Make Your Transplant Happen” (pages 10 to 12)  <https://unos.org/wp-content/uploads/Brochure-106-Find-living-donor.pdf> |
| UNOS Ambassador Program | <https://unos.org/community/unos-ambassadors/> (Requires an account, free) |
| American Kidney Fund (AKF) | “How to Ask for a Kidney Donation”  <https://www.kidneyfund.org/kidney-donation-and-transplant/how-ask-kidney-donation> |
| American Liver Foundation | “Sharing your story on social media: how to use Facebook” https://liverfoundation.org/resource-center/documents/sharing-your-story-on-social-media-how-to-use-facebook/ |

Supplement 1. List of resources publicly available (non-comprehensive) with guidance on using social media to identify potential living donors.
